# Chemical Cues Influence Pupation Behavior of *Drosophila simulans* and *Drosophila buzzatii* in Nature and in the Laboratory

**DOI:** 10.1371/journal.pone.0039393

**Published:** 2012-06-21

**Authors:** Marcial Beltramí, María Cristina Medina-Muñoz, Francisco Del Pino, Jean-Francois Ferveur, Raúl Godoy-Herrera

**Affiliations:** 1 Departamento de Biología, Facultad de Ciencias Naturales y Exactas, Universidad Metropolitana de Ciencias de la Educación, Santiago, Chile; 2 Departamento de Biología, Facultad de Ciencias Naturales y Exactas, Universidad de Playa Ancha de Ciencias de la Educación, Valparaíso, Chile; 3 Programa de Genética Humana, ICBM, Facultad de Medicina, Universidad de Chile, Santiago, Chile; 4 Centre des Sciences du Gout et de l’Alimentation, Unité Mixte de Recherche 6265 Associée au Centre National de la Recherche Scientifique, Université de Bourgogne, Faculté des Sciences, Dijon, France; 5 Departamento de Ciencias Básicas, Facultad de Ciencias, Universidad del Bío-Bío, Campus Fernando May, Chillán, Chile; Center for Genomic Regulation, Spain

## Abstract

In the wild, larvae of several species of *Drosophila* develop in heterogeneous and rapidly changing environments sharing resources as food and space. In this scenario, sensory systems contribute to detect, localize and recognize congeners and heterospecifics, and provide information about the availability of food and chemical features of environments where animals live. We investigated the behavior of *D. simulans* and *D. buzzati*i larvae to chemicals emitted by conspecific and heterospecific larvae. Our goal was to understand the role of these substances in the selection of pupation sites in the two species that cohabit within decaying prickly pear fruits (*Opuntia ficus-indica*). In these breeding sites, larvae of *D. simulans* and *D. buzzatii* detect larvae of the other species changing their pupation site preferences. Larvae of the two species pupated in the part of the fruit containing no or few heterospecifics, and spent a longer time in/on spots marked by conspecifics rather than heterospecifics. In contrast, larvae of the two species reared in isolation from conspecifics pupated randomly over the substrate and spent a similar amount of time on spots marked by conspecifics and by heterospecifics. Our results indicate that early chemically-based experience with conspecific larvae is critical for the selection of the pupation sites in *D. simulans* and *D. buzzatii*, and that pupation site preferences of *Drosophila* larvae depend on species-specific chemical cues. These preferences can be modulate by the presence of larvae of the same or another species.

## Introduction

*Drosophila* adult and larva behaviors in nature have been poorly investigated [1], [2]. This causes a limitation in the investigation related to population genetics, evolution and neurosciences in *Drosophila* genus. For example, even if we know that *Drosophila* larvae can learn [3], the role of learning in adaptation to changing breeding sites, where these pre-adults develop, remains poorly explored. This knowledge is important to fully understand population genetics and ecology in relation to the evolution of *Drosophila* genus. In particular, we have very little knowledge on the involvement of learning in food preference, feeding rate, and selection of pupation sites. Since many *Drosophila* larvae develop within decaying fruits, which change their composition in a relatively short time, *Drosophila* larval behavior may show a high flexibility and plasticity [4]. On the other hand, ecological variation of the breeding sites also may affect some aspects of *Drosophila* adult life cycle, as the reproduction strategies [5] and selection of oviposition sites [6].

Even if the cellular and molecular genetics bases of odor perception have been intensively unraveled in *Drosophila* adults and larvae [7], the identity of natural chemicals surrounding the breeding sites is virtually unknown. Several studies suggested that the selection of pupation sites in species of *Drosophila* breeding in the same breeding sites largely depend on the discrimination of chemicals emitted by conspecifics and heterospecifics [1], [8]. Here, we focused on the selection of pupation sites by larvae of *Drosophila simulans* (Subgenus *Sophophora; melanogaster* species group) and *Drosophila buzzatii* (Subgenus *Drosophila; repleta* species group) in nature and in the laboratory. In Chile, the two species together with *Drosophila nigricruria* and *Drosophila hydei* (Subgenus *Drosophila*; *repleta* species group) cohabit in decaying prickle pear fruits (*Opuntia ficus-indica*) and necrotic tissues of this plant and columnar cactus *Echinopsis chilensis*. In Argentina, *D. buzzatii* lives in desertic and woodland areas in association with one or more species of *Opuntia*. Thus, *D. buzzatii* is a widespread species with colonizing ability. *Drosophila* pupation behavior is related with habitat selection, colonization of new niches and the expansion of populations [9], [10]. Specifically, we focused on the natural phenotypic variation in pupation site preferences resulting of the presence of homo- and heterospecific individuals sharing the same breeding site. For each species, we tested the effect of larval chemicals on the selection of pupation site.

## Results

### Natural Variation in Pupation Site Preferences

**Figure 1:**
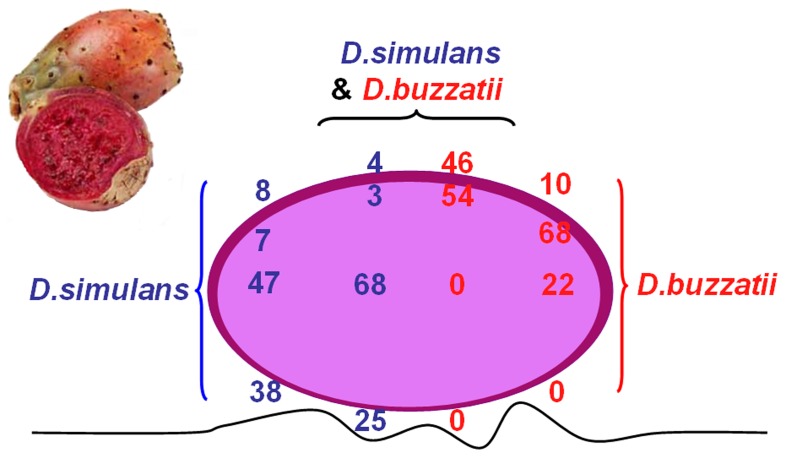
Distribution (percentages) of *D. simulans* and *D. buzzatii* pupae on decaying prickly pear fruits. The pupae were detected inside the fruit, under the skin, over the skin and under the fruit on the land (convoluted line). Number of fruits (N = 86) collected with pupae of the two species: *D. simulans* pupae (N = 671); *D.* *buzzatii* pupae (N = 380). Number of fruits (N = 46) collected with only *D. simulans* pupae (N = 521). Number of fruits (N = 47) collected with only *D. buzzatii* pupae (N = 432).

In their natural breeding sites, *D. simulans* and *D. buzzatii* changed their pupation site preferences in the presence of the other species. In the absence of *D. buzzatii*, about half *D. simulans* larvae pupated inside the fruit (Fig. 1), and most of the other pupae were found on the land underneath the fruit (*D. simulans* pupae were rarely found near-under and over-the fruit skin). When mixed with *D. buzzatii*, most *D. simulans* pupae developed inside the fruits, whereas fewer were found on the land underneath the fruits or in contact with the fruit skin. Therefore, the presence of *D. buzzatii* changed *D. simulans* pupation site preference in the nature, Fig.1; χ^2^ = 117.16, *df*  = 4, *P*<<0.01.

Reciprocally, in the fermented fruits only colonized by *D. buzzatii*, most pupae were found under the skin, whereas the remaining pupae were found inside the fruit and much frequently on the skin surface. In the presence of *D. simulans*, most *D. buzzatii* pupae were found –under or over- the skin. This effect was highly significant, Fig.1; χ^2^ = 18.29, *df*  = 2, *P*<<0.01. In conclusion, the presence of heterospecifics affected the distribution of pupae in different microhabitats of the fruit.

### Larval Behavior to Conspecific and Heterospecific Larval Chemical Cues

**Figure 2:**
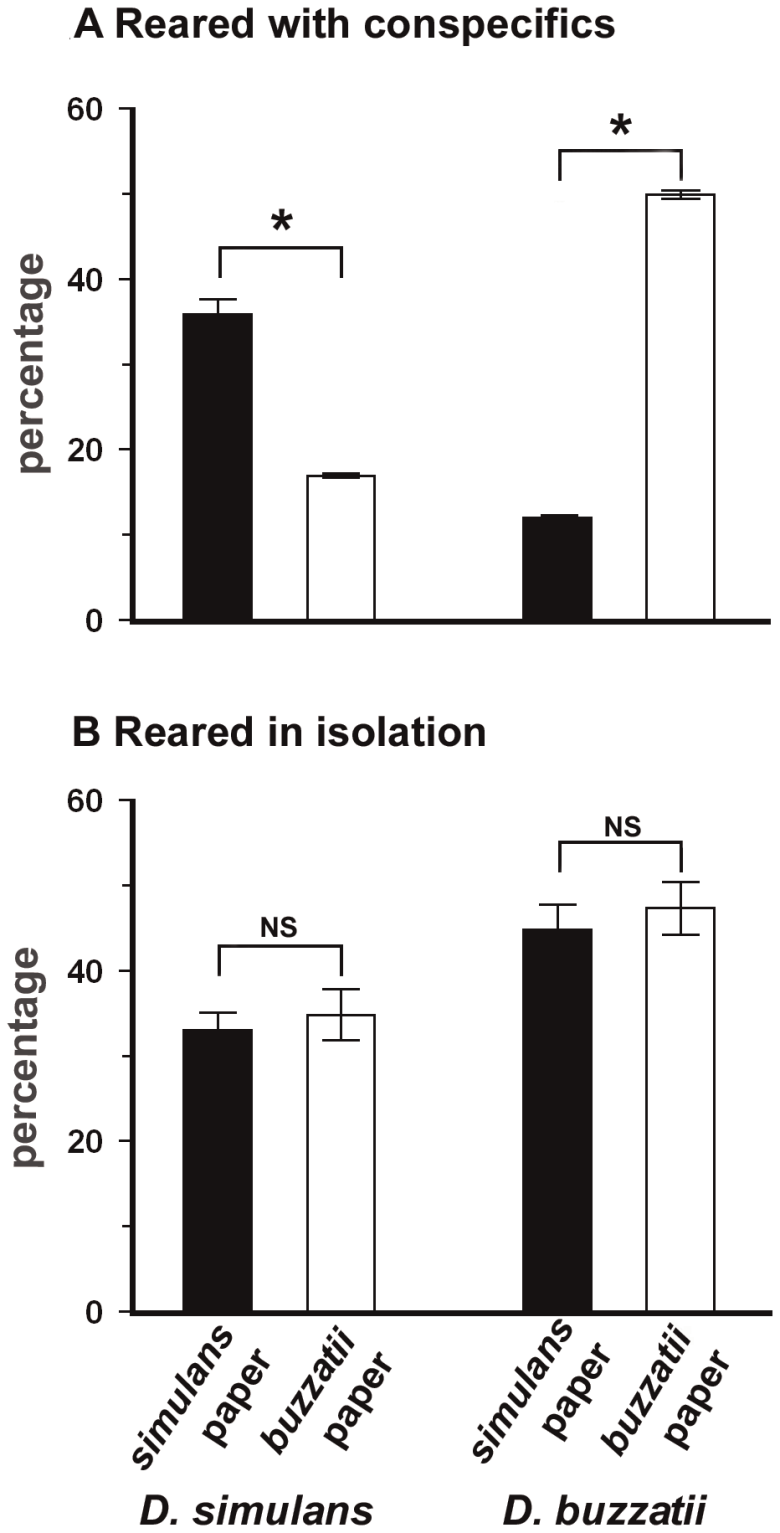
Time, min; mean ± S.E., spent by third instar larvae of *D. simulans* and *D. buzzatii* on conspecific and heterospecific papers. The larvae were reared with conspecifics (Fig. 2a), and in isolation from conspecifics (Fig. 2b), and tested individually. Black columns, time spent on *simulans* paper; white columns time spent on *buzzatii* paper. *Mann-Whitney *U*-test; P<0.05; NS  =  non significant differences (see text for details).

Since our observation in nature indicated that interaction between larvae of the two species can affect their pupation site, back in the laboratory, we tested the role of chemical cues potentially involved in this effect. To asses this, we measured both the larval behavior and pupation site preferences in larvae presented to a dual-choice test consisting of two filter paper targets impregnated by the food processed by larvae of each species. First, we measured the time spent by third instar *D. simulans* and *D. buzzatii* larvae on each target paper. The performance of larvae either reared with conspecifics or in isolation was compared (Fig.2). *D. simulans* larvae reared with conspecifics remained much longer on the *D. simulans* paper than on the *D. buzzatii* paper (15.6 and 5.0 min, respectively; *U*_0.05, 5,19_ = 78; *P*<0.05; Fig. 2a). Reciprocally, *D. buzzatii* larvae reared with conspecifics spent much more time on the *D. buzzatii* paper than on the *D. simulans* paper (10.3 and 3.0 min, respectively; *U*_0.05, 6,12_ = 61; *P*<0.05). By contrast, larvae of the two species reared in isolation showed no marked preferences: they spent a similar amount of time on the *D.* *simulans* and *D. buzzatii* papers (for *D.* *simulans* larvae: 8.7 and 10.5 min, respectively; *U*_0.05, 4, 6_ = 2.0; *P>*0.05; for *D. buzzatii* larvae: 6.7 and 7 min, respectively; *U*_0.05, 2, 16_ = 3.6; *P>*0.05; Fig. 2b). In conclusion, this experiment shows that *D. simulans* and *D. buzzatii* larvae raised with conspecifics spent more time on sites impregnated with their own species-specific chemicals.

### Influence of Conspecific Larval Chemical Cues on Pupation Site Preferences

**Figure 3:**
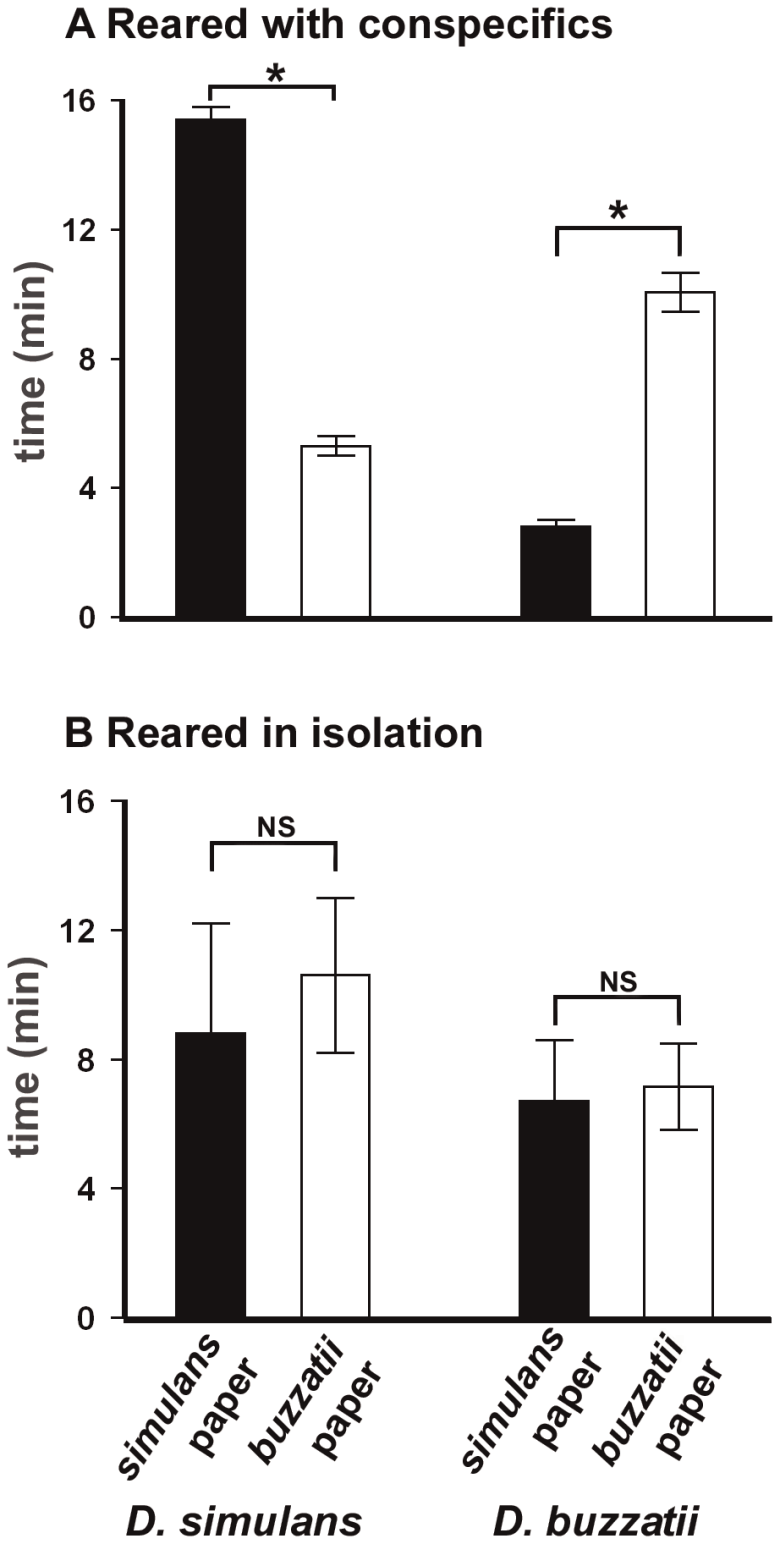
Mean ± S.E., of pupae of *D. simulans* and *D. buzzatii* on conspecific and heterospecific papers. The larvae were of third instar reared with conspecifics (Fig. 3a), and in isolation from conspecifics (Fig. 3b). The larvae were tested individually (N  = 50 per species). Black columns, percentages of pupae on the *simulans* paper; white columns percentages of pupae on the *buzzatii* paper. *Mann-Whitney *U*-test; P<0.05; NS  =  non significant differences (see text for details).

We also studied the effect of conspecifics on pupation site preferences by comparing the performance of individuals raised either alone or with conspecifics (Fig. 3). *D. simulans* larvae raised with conspecifics pupated much more often on *D. simulans* target paper (35.8%, than on *D. buzzatii* paper, 10.2%, *U*_0.05, 10, 30_ = 221; *P*<0.05). Similarly, *D. buzzatii* larvae raised with conspecifics pupate preferentially on the conspecific paper than on the *D. simulans* paper (49.0 and 12.0%, respectively; *U*_0.05, 10, 35_ = 249; *P*<0.05. By contrast, larvae reared in isolation showed no pupation site preference. More precisely, a similar frequency of pupae was observed on either *D. simulans* or *D. buzzatii* papers (for *D. simulans*: 32.2 and 33.8%, respectively; for *D. buzzatii*: 42.1 and 46.3%, respectively). In conclusion, *D. simulans* and *D. buzzatii* third instar larvae raised with conspecifics distinguished *simulans* and *buzzatii* chemical cues to select pupation sites, but they did not when bred in isolation. These results suggest that larval experience with conspecifics play a critical role in pupation site preferences in both species.

## Discussion

Larvae of a wide range of *Drosophila* species use the same decaying fruits as breeding sites [11]. This implies that they are adapted to grow and develop in the presence of other species of the genus, and this may be reflected by their responses to chemicals emitted by other larvae. Our data support this assumption; *D. simulans* and *D.* *buzzatii* larvae responded to chemical cues produced by individuals of the two species. As a result the larvae changed their pupation site preferences. Most importantly, larvae of each species pupated away from the pupation sites selected by larvae of the other species. These findings are supported by previous studies [1], [8], suggesting that the pupation behavior shown by *D. simulans* and *D. buzzatii* is part of larval behaviors that allow the optimal exploitation of breeding site units by *Drosophila* species.

On the other hand, responses to chemical cues produced by *D. simulans* and *D.* *buzzatii* larvae introduce a spatial dimension to pupation behavior. This means that the larval selection of pupation sites may result in space competition within the breeding sites, this affecting the expansion rate and dynamic of the populations. In other words, the spatial localization of *D. simulans* and *D. buzzatii* pupae within their breeding sites may influence the ecological, genetic and evolutionary dynamics of their populations [2], [12].

Our data also show that rearing conditions change the dispersal patterns of *D. simulans* and *D. buzzatii* larvae prior to their selection of pupation sites. The fact that larvae reared alone spent less time on a paper impregnated in conspecific chemical cues than larvae of the same strain reared with conspecifics (Fig. 2 a, b) suggests that early experience with conspecifics will subsequently affect the patterns of pupae. Given the heterogeneous and changing ecological conditions of decaying prickly pear fruits where larvae develop, the detection, recognition, and spatial discrimination of conspecific and heterospecific cues may help dispersing larvae to find their way within the breeding sites. On the other hand, larval behaviors are flexible, and this flexibility depends on species, rearing conditions during early development, and ecological circumstances including the presence/absence of heterospecifics (Figs. 1–3). Then, Drosophila breeding sites provide environments where recognition of chemical cues left by conspecifics maybe crucial for the selection of pupation sites. Our data also suggest that, in the wild, stimuli provided by conspecifics and heterospecifics are processed through some type of learning process as described in *D. melanogaster* larvae [3], [13], [14]. Further work is required to discover the mechanisms and neural pathway involved in this process.

Our study shows that pupation site preferences of *Drosophila* larvae depend on species-specific chemical cues. These preferences can be changed by the presence of other larvae of the same or of another species. Our data suggest that the natural sites where *D. simulans* and *D. buzzatii* pupae are found reflect a careful choice of the most appropriate place to pupate. Similar studies could help to investigate the role of biotic factors in other ecologically relevant larval behaviors such as feeding rates and food preferences. Our study should also provide a new insight to understand how the choice of niche can be modulated through the neural perception of chemical cues affecting pupation behavior on a short term, and perhaps driving evolutionary changes on the long term. The nature of these signals is unknown, but *Drosophila* larvae could produce pheromones as those involved in mate recognition in the *melanogaster* Subgroup [9]. Clearly, future work should focus on this problem.

## Materials and Methods

### Field Collection and Strains

We used both cosmopolitan *D. simulans* (subgenus *Sophophora*; *melanogater* species group) and widespread *D. buzzatii* (subgenus *Drosophila*; *repleta* species group). In Chile, the two species have a strong colonizing ability. Thus, larvae, pupae and adults can be collected from decaying fruits of prickly pear *Opuntia ficus-indica* between Copiapó, in the northern part of Chile (latitude 25° 15′), and Curicó, in the south (latitude 34° 38′). Some very few adults of *D. melanogaster*, *D. nigricuria*, *D. hydei* and Chilean endemic *D. pavani* may also emerge from the fruits and necrotic prickly pear tissue. The fruits containing pupae of *D. simulans* and *D. buzzatii* were collected in Til-Til (latitude 33° 5′), 50 km North-East from Santiago in April of 2008 when Chilean populations of *Drosophila* reach their peak of abundance [15]. Thus, we used sympatric strains of the two species. Fruits were randomly picked up. We collected 46 fermented fruits of prickly pear containing pupae of *D. simulans*, other 47 with pupae of *D. buzzatii*, and 86 fruits with pupae of both species. We counted the number of pupae on different parts of the fruits (inside the fruit tissue, over the skin, under the skin) and on the land underneath the collected fruits (Fig. 1). Species were identified on the base of the color and size characteristics of pupae [1]. For the few unidentified cases, each pupa was individually deposited in a vial containing a piece of moistened filter paper, and stored in an incubator at 24°C until emergence for species identification. *D. simulans* and *D. buzzatii* studied lines were initiated with flies emerging from fermented prickle pear fruits (details in [1]). All lines were maintained at 24°C in half pint bottles containing 50 cc of [16].

### Laboratory Tests

To measure pupation site preference and larval behavior, third instar larvae were individually placed at the center of a Petri dish filled with 3% agar with a thickness of 2 cm. On both sides of the center (at about 3 cm) of the dish, we placed two round pieces of filter paper (diameter  = 2 cm). Each piece of paper was impregnated during 1 h in Burdick’s medium processed by larvae of either species. For larval behavior experiment, we recorded the time spent on each type of impregnated paper by individual larva (N  = 50/species). All these larvae were reared together in Burdick’s medium. We also measured the behavior of 50 other individuals of the same species reared in isolation from conspecifics. After larval behavior measurement, the Petri dishes containing the impregnated papers were incubated at 24°C until pupation and the number of pupae found on each type of paper was noted (pupae found under, around and under the paper were pooled).

### Statistical Analysis

To compare the distributions of pupae on different parts of the fruit, we used R x C test of independence. We used the Mann-Whitney test to compare time of permanence, and pupation site preferences of the larvae reared with/out conspecifics [17].

No specific permits were required for the described field studies from any authority. We talked with the owner of the private land, “Fundo La Capilla", where the field studies were made, Don Juan Ignacio Herrera and Don Gonzalo Herrera, who agreed we examined, collected decaying fruits, and the fruit flies for our field and laboratory studies. The fruit units examined were fallen on the ground; they correspond to disposable fruits unfit for human consumption. The field studies did not involve endangered or protected species.
